# Evaluation of the effect of soft drinks on the surface roughness of dental enamel in natural human teeth

**DOI:** 10.12688/f1000research.55556.1

**Published:** 2021-11-10

**Authors:** Ibrahim Al-Amri, Roula Albounni, Sultan Binalrimal

**Affiliations:** 1 Restorative Dentistry, Riyadh Elm University, Riyadh, 13781, Saudi Arabia

**Keywords:** Soft drinks, surface roughness, dental enamel, pH

## Abstract

**Background**: Exposing enamel to soft drinks and beverages causes changes in the microscopical morphology. Changes in the physical characteristics, like hardness and surface roughness, were studied with various parameters. Factors such as pH, exposed duration, and other content of the soft drinks have different effects on the enamel.

**Methods**: Thirty-six human premolar teeth were randomly divided into three groups (n=12). Group 1 consisted of teeth which were immersed in artificial saliva (control), group 2 consisted of teeth which were immersed in Pepsi, and group 3 consisted of teeth which were immersed in Mirinda. After the preparation of samples and necessary measures, surface roughness was measured using the profilometer. Baseline measurements were taken. Reading two and reading three were taken after exposing the specimens for three days and 15 days, respectively. All of the collected data were compared statistically using SPSS and presented in tables and graphs.

**Results: **At the baseline, the surface roughness value was the same for all three groups. On the 3 ^rd^ day, the Mirinda group showed more surface roughness compared to that of the test and Pepsi groups, which was statistically significant. On the 15 ^th^ day, both the Pepsi and Mirinda groups showed increased surface roughness compared to that of the control, which was statistically significant.

**Conclusions: **Within the limitation of this study, it can be concluded that surface roughness increased when teeth were exposed to both soft drinks

## Introduction

Modern-day food and drink has a deleterious effect on the oral cavity including the tooth; though enamel is considered to be the hardest part of the body, exposure to many foods and beverages has been shown to have a negative impact on the enamel. ^1^ Though the enamel surface appears to be flat, it is not. It has a wavy structure because at places where Retzius’ striae end such striae overlap in the form of steps, with the appearance of shallow grooves referred to as perikymata. With its organic and inorganic structure, enamel shows pronounced hardness, but it is also fragile at the same time and similar to glass. Exposure to several environmental factors including food and beverages makes this strongest part of the body susceptible to breakdown. ^2^

Tooth wear is thus considered as an additive, multifactorial lifelong event which is of great concern because it is irreversible. ^3^ Among these, tooth erosion has taken a major toll on management of tooth wear. Tooth substance loss associated with erosion by acidic foods and soft drinks is an increasing social problem because it is frequently linked with individuals’ lifestyle and eating habits. ^1^ Consumption of these beverages and soft drinks has increased worldwide, especially in the younger generation, which leads to the early loss of teeth, and is a serious concern. This impact on oral health, and teeth as such, has led to much research to find out the exact factors associated with this and thus how to overcome this effect. ^4^

In the modern world, with the influence of the western world, this increased consumption of soft drinks and carbonated beverages is becoming a problem. These soft drinks are becoming popular because of added content to these drinks, which increase the palatability or taste. The major added content to these soft drinks is citric acid, phosphoric acid, and malic acid. ^5^ If the soft drink is the external factor for the alteration in tooth enamel to occur, there are also intrinsic factors, such as eating disorders or bulimia and/or gastric reflux, which result in the output of gastric content into the oral cavity. This leads to a loss of tooth substance or erosion of the tooth on the lingual aspect of the teeth, sparing the buccal aspect. ^1^

Within the available literature, it was found that several factors influence the behavior of enamel when it is exposed to soft drinks and beverages. These factors are pH, acidic species, calcium and phosphate contents, and exposure time. ^1^^,^^6^^,^^7^ For enamel demineralization to occur, the pH on the enamel surface must fall below 5.5. Many available soft drinks have a pH value well below 5.5. Several studies ^3^^,^^4^ have been published on the pH values of soft drinks, as an indicator of their erosive potential; though it is an important component of the soft drink to be related to the erosion, the response can be modified by other factors like calcium and phosphate content and the amount of time the enamel is in contact with the soft drinks. ^1^

To date, many studies have mainly concentrated on two factors, pH and the physical characteristics of the enamel. This property has been evaluated by measurement of the roughness of enamel. ^4^^,^^8^ Certain quantitative evaluations, using various devices like the profilometer, have been the standard for research activities which define the loss of substance which occurs due to exposure to soft drinks. However, many studies lack an explanation for the time-bound exposure of these agents and their effect on loss of tooth substance. Thus, the present study is undertaken to investigate the effect of soft drinks on the surface roughness of dental enamel and compare the effect of two kinds of soft drinks (Pepsi and Mirinda) on the surface roughness of dental enamel in two time periods.

## Methods

A total of 36 extracted human premolar teeth, which were extracted for orthodontic treatment at the dental hospital of Riyadh Elm University, were taken. The study was approved by the Institutional Review Board (IRB) of Riyadh Elm University (IRB approval number FPGRP/2020/516/304/300) and consent was waived by the committee. Teeth were carefully cleaned with distilled water and a soft brush then stored in distilled water. Teeth were sectioned, first longitudinally from mesial to distal of the central fissure, and second transversely 2mm below the cementoenamel junction (CEJ) using a slow speed saw machine with a diamond disc to separate the buccal surface of the tooth. The buccal surfaces were then embedded in acrylic resin to keep them free from acrylic resin in order to obtain the surface roughness. After placing all samples in acrylic resin mold, teeth were randomly divided into three groups:

Group 1 (n = 12): Teeth immersed in 150 ml artificial saliva (control group) (the composition of the artificial saliva was a combination of water and the following: carboxymethylcellulose [CMC], glycerin, minerals such as phosphates, calcium, fluoride, and xylitol. It also contains preservatives to maintain shelf life and flavoring agents to give them a pleasant taste). ^9^

Group 2 (n = 12): Teeth immersed in 150 ml of Pepsi (the composition of Pepsi is carbonated water, sugar, color [caramel E150d], acid [phosphoric acid] and flavorings [including caffeine]).

Group 3 (n = 12): Teeth immersed in 150 ml of Mirinda (the composition of Mirinda is carbonated water, high fructose corn syrup and/or sugar, citric acid, purity gum, potassium benzoate and potassium sorbate [preserves freshness], ester gum, natural flavor, yellow 6, ascorbic acid and calcium disodium EDTA [to protect flavor] and sodium citrate).

The control groups were immersed in artificial saliva, the test soft drink groups (Pepsi and Mirinda) were changed daily, and the samples were immersed at 37° C. Baseline measurements for all of the samples were taken with the profilometer, and the baseline data was recorded in a special form to be analyzed later.

A respective number of samples were immersed in the control solution and two test beverages according to the aforementioned division of all samples, for two periods of time, first for three days and then for 15 days in the second round. After each period all samples were washed with distilled water then subjected to roughness measurements using the same device, then all results were recorded in the same form for each group. Characterization and imaging were performed using a Contour GT-K 3D Optical Microscope (Bruker ^®^), and 3D non-contact surface metrology with interferometry. A 5× Michelson magnification lens with a field of view of 1.5 × 1.5 mm, a Gaussian Regression Filter, a scan speed of 1×, and thresholding of 4 was set for this study.

Samples were placed on the stage and manually adjusted to give an image on the monitor screen. The microscope uses Vision 64 (Bruker ^®^) software which controls the instrument settings, data analyses, and graphical output. The measurement was performed using vertical scanning interferometry which uses a broadband (normally white) light source which is effective for measuring objects with rough surfaces, as well as those with adjacent pixel-height differences greater than 135 nm. Each sample was scanned at three intervals and averaged accordingly to determine the roughness value.

Descriptive statistics such as mean and standard deviation were used. One-way repeated measurement ANOVA (analysis of variance) or two-way ANOVA was used to compare the effect of solutions and time by the average Ra. Tukey’s multiple comparison test was used when ANOVA showed a significant difference.

### Data analysis

Data was analyzed using statistical package for the social sciences (SPSS) version 20.0 (SPSS Inc., Chicago, II, USA). A p-value of ≤0.05 was considered statistically significant.

## Results

The analysis showed there was a significant interaction between the reading factor and solution factor (p < 0.0001) ( Table 1). The mean ± sd baseline readings were 1.097 ± 0.245, 1.187 ± 0.260, and 1.187 ± 0.255 for the control group, Pepsi, and Mirinda, respectively. The ANOVA shows that there was no significant difference among solutions in their roughness means (p = 0.634).

**Table 1.** Tests of within-subject effects.

Measure						
Source		Type III sum of squares	df	Mean square	F	Sig.
Reading	Sphericity assumed	10.253	2	5.127	23.64	0
	Greenhouse-Geisser	10.253	1.429	7.174	23.64	0
	Huynh-Feldt	10.253	1.566	6.546	23.64	0
	Lower-bound	10.253	1	10.253	23.64	0
Reading * solution	Sphericity assumed	5.273	4	1.318	6.079	0
	Greenhouse-Geisser	5.273	2.858	1.845	6.079	0.002
	Huynh-Feldt	5.273	3.133	1.683	6.079	0.001
	Lower-bound	5.273	2	2.637	6.079	0.006
Error (reading)	Sphericity assumed	14.313	66	0.217		
	Greenhouse-Geisser	14.313	47.165	0.303		
	Huynh-Feldt	14.313	51.691	0.277		
	Lower-bound	14.313	33	0.434		

Additionally, within reading two, the solutions’ mean ± slandered deviation of roughness was 1.339 ± 0.209 for control, 1.580 ± 0.249 for Pepsi, and 1.90 ± 0.330 for Mirinda. However, ANOVA showed that at least one of the solutions was significantly different in roughness from the other (p < 0.001). Tukey’s multiple comparison test indicated that the Mirinda group had significantly higher mean roughness than the Pepsi and control groups (p < 0.05). Also, the Tukey post hoc test did not find a significant difference between the average roughness of the control and Pepsi groups (p = 0.085).

**Figure 1.**
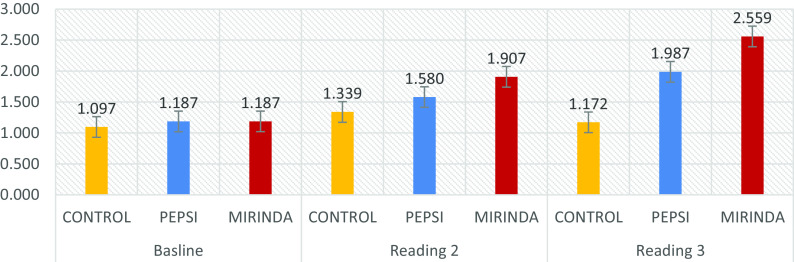
Solution with each surface roughness read.

However, within reading three the solutions’ mean readings were 1.172 ± 0.425, 1.987 ± 0.832, and 2.559 ± 0.783 for control, Pepsi, and Mirinda, respectively. ANOVA showed that at least one of the solutions was significantly different in terms of mean roughness from the others (p < 0.001). As a consequence, the multiple compression test indicated that the Pepsi and Mirinda groups had significantly higher mean roughness than the control group (p < 0.05), and there was no significant difference between the average roughness of the Pepsi and Mirinda groups (p = 0.139) ( Table 2 and Figure 1).

**Table 2.** Comparison of the surface roughness among different groups.

Reading	Solution	N	Mean	Std. deviation	Analysis of variance (ANOVA) p-value	95% confidence interval for mean		Tukey as multiple comparison test		
						Lower bound	Upper bound	CONTROL	PEPSI	MIRINDA
Baseline	CONTROL	12	1.097	0.246	**0.634**	0.941	1.253	1		
	PEPSI	12	1.187	0.260		1.021	1.352	NS	1	
	MIRINDA	12	1.187	0.255		1.025	1.349	NS	NS	1
Reading 2	CONTROL	12	1.339	0.209	**0.000**	1.206	1.472	1		
	PEPSI	12	1.580	0.249		1.422	1.738	0.085	1	
	MIRINDA	12	1.907	0.330		1.697	2.116	**0.000**	**0.014**	1
Reading 3	CONTROL	12	1.172	0.425	**0.000**	0.902	1.442	1		
	PEPSI	12	1.987	0.832		1.459	2.516	**0.021**	1	
	MIRINDA	12	2.559	0.788		2.059	3.060	**0.000**	0.139	1

One-way ANOVA was used to study the effect of reading or time by comparing the roughness average within each solution. However, within the control solution the reading means were 1.097 ± 0.246, 1.339 ± 0.209, and 1.172 ± 0.425 for the baseline reading, reading two, and reading three, respectively. ANOVA showed no significant difference among readings in terms of roughness (p = 0.158).

Additionally, within the Pepsi group, the readings of roughness were 1.187 ± 0.260 for the baseline reading, 1.580 ± 0.249 for reading two, and 1.987 ± 0.332 for reading three, However, ANOVA showed that at least one of the readings was significantly different in roughness from the others (p < 0.001). Furthermore, the multiple compression test indicated that reading three has significantly higher mean roughness than the baseline reading (p < 0.05), but there was no significant difference between the average roughness of reading two and reading three (p = 0.153).

**Figure 2.**
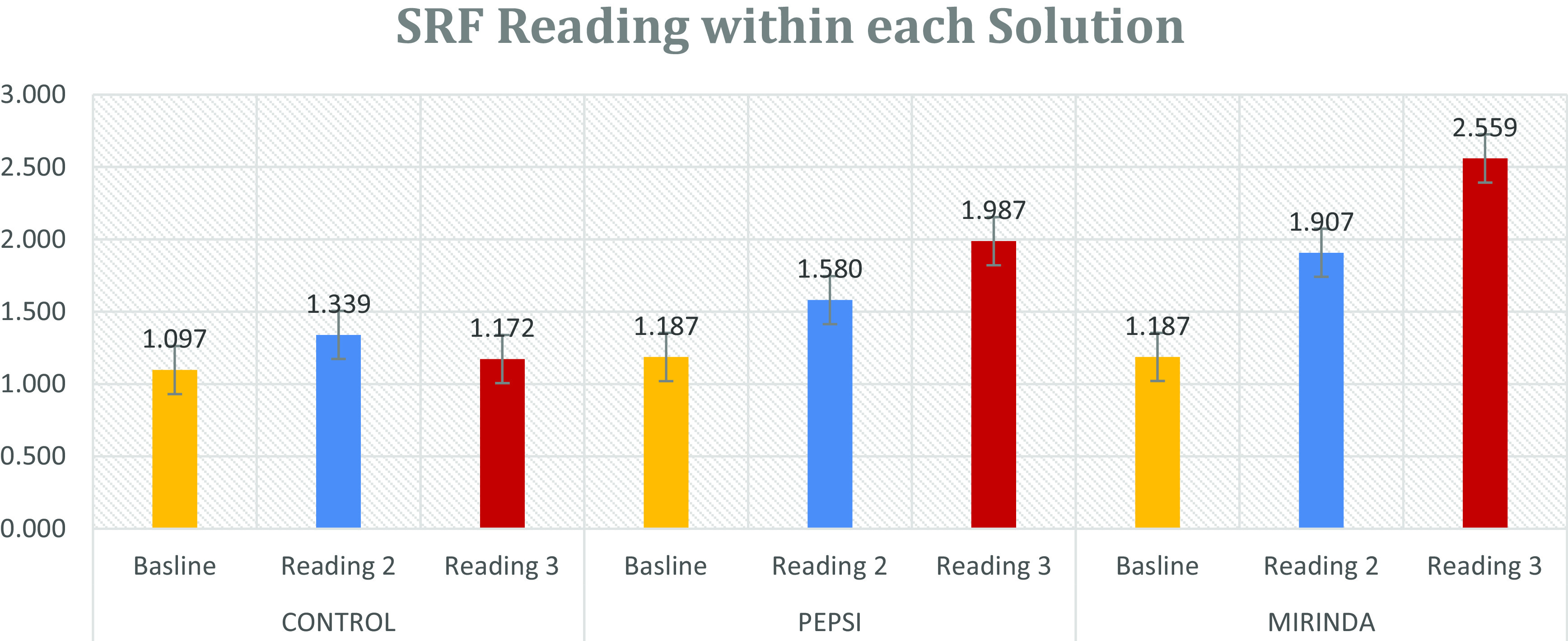
Surface roughness reading within each solution.

Moreover, within the Mirinda solution, the reading means were 1.187 ± 0.255, 1.967 ± 0.330, and 2.559 ± 0.780 for the baseline, reading two, and reading three, respectively. ANOVA showed that at least one of the readings was significantly different to the others in terms of roughness (p < 0.001). As a result, the multiple compression test indicated that reading two and reading three had significantly higher roughness than the baseline reading (p < 0.05), and there was no significant difference between the roughness of reading two and reading three (p = 0.139) ( Table 3 and Figure 2).

**Table 3.** Comparison of the surface roughness within each group.

Solution	Reading	N	Mean	Std. deviation	Analysis of variance (ANOVA) p-value	95% confidence interval for mean		Tukey as multiple comparison test		
						Lower bound	Upper bound	CONTROL	PEPSI	MIRINDA
CONTROL	Baseline	12	1.097	0.246	**0.158**	0.941	1.253	1		
	Reading 2	12	1.339	0.209		1.206	1.472	NS	1	
	Reading 3	12	1.172	0.425		0.902	1.442	NS	NS	1
PEPSI	Baseline	12	**1.187**	0.260	**0.003**	1.021	1.352	1		
	Reading 2	12	1.580	0.249		1.422	1.738	0.172	1	
	Reading 3	12	**1.987**	0.832		1.459	2.516	**0.002**	0.153	1
MIRINDA	Baseline	12	1.187	0.255	**0.000**	1.025	1.349	1		
	Reading 2	12	1.907	0.330		1.697	2.116	**0.021**	1	
	Reading 3	12	2.559	0.788		2.059	3.060	**0.000**	0.139	1

## Discussion

In modern society, consumption of carbonated beverages is so common that it has replaced drinking water, which has led to many ill effects and has been discussed in many studies. ^1^ Enamel, though the hardest part of the body, is still a vulnerable structure when exposed to chemicals, like those found in soft drinks and beverages. Among the factors which are associated with this are pH and other factors like calcium and the phosphate content of the soft drinks. Changing the viscosity has shown a positive effect and reduction in the erosion of the enamel. ^10^

The present study utilized the commonly consumed beverages in the Saudi market which are Pepsi and Mirinda. Timed exposure to the test specimens and artificial saliva was taken, with three days and 15 days as a time interval which will help to co-relate the timed exposure that may occur in the oral cavity. Surface roughness is one of the parameters used to assess the effect of soft drinks on enamel. Other aspects like hardness also have been studied. However, surface roughness is the initial change in the enamel that appears due to exposure to soft drinks and beverages. To measure this a profilometer, which has been commonly used in many previous studies, has been utilized. ^4^^,^^11^

The surface roughness of all the samples measured at the baseline showed no difference. However, the samples compared at three days have shown that the soft drink Mirinda causes a comparatively rougher surface than the other substances. These results are similar to the results of previous studies. The reason for this could be due to the composition of Mirinda compared to Pepsi. It is very well known that the most common type of acid used in soft drinks is citric acid, which has greater erosive potential. Citric acids and/or citrates are added as buffering and flavoring agents, but they can concurrently bind to calcium and phosphorus thereby promoting increased titratable acidity levels. ^12^

It was shown that most carbonated beverages have a pH of 2.6. ^3^ However, the actual pH may vary in different soft drinks (which is the trade or brand secret) and may lead to surface changes seen which are different in three days compared to the Pepsi and control groups. One earlier study has shown that Coca-Cola has the lowest pH of all the test drinks, so it shows the highest change in surface roughness. ^13^ A study where they have compared cola and orange juice has shown that the eroded surfaces in cola and orange juice groups were visibly roughened and had lost their luster as compared to the control surface. ^11^ Similarly, Barac *et al.* also showed results similar to the present study. ^11^ In the present study roughness was measured at the end of the third day, while the above studies have had exposure times of 30 minutes and 60 minutes. Though there is a time difference, the results of the study have shown the same outcome with all the drinks showing surface roughness.

In the present study surface roughness was assessed again on the 15th day. The results of the study have shown that both the test groups, Pepsi and Mirinda, showed higher roughness compared to the control group. Again, these results were similar to previous studies. Trivedi *et al*. measured surface roughness at the 14 day point in their study, and the high-energy sports drink and non-carbonated beverage showed a highly significant difference compared to the control. ^12^ Similarly, an in vitro study by Navarro *et al.* which measured bond strength, bracket microleakage, and adhesive remnant on intact and sealed enamel in 15 days also resulted in more roughness and a change in the hardness of the enamel, which led to the decreased bond strength and microleakage. ^14^ Thus, the present study results are comparable to the previous results.

In the present study, it was found that there was no difference in surface roughness at day 15 for both the Pepsi and Mirinda groups. These results are similar to the study done by Barac *et al.* ^11^ They found that roughness parameters with Coca-Cola had the strongest erosion potential during the 15 min of exposure, while Coca-Cola and orange juice showed similar results during 30- and 60-min exposures. There was no difference between 60 minutes and 10 days’ exposure. However, this result was in contrast to the study by Rajeev and Lewis, where they found that the maximum roughness was seen at 10 days with lime juice and lime soda. The difference in the result is due to the variations in the beverages and amount of exposure to these beverages. ^3^

A change in surface roughness in the hardness of the enamel is common after exposure to soft drinks. This has been proven in the current and previous studies. To overcome and reduce the effect of soft drinks, many measures and treatment modalities have been attempted. All studies have shown favorable results. They have used fluoride, casein phosphopeptide-amorf calcium phosphate, and acidulated phosphate fluoride gel, ^15^ CPP-ACPF and nano-hydroxyapatite, ^16^ air-abrasion pre-treatment with bioactive glass 45S5, ^17^ and many other treatments. These research reports show the seriousness of the surface roughness problem associated with soft drinks.

This study report, with three days and 15 days of exposure, shows that one needs to be well aware of the effect of soft drinks on enamel. Dentists need to educate patients regarding these soft drinks and ways to reduce the exposure to them. The limitations are, mimicking the oral mouth environment and salivary pH which affect the roughness property strongly, compositions of the enamel and its properties differ between vital and non-vital teeth, and personal oral hygiene and dental habits could be an important factor that affects the result.

## Conclusions

Within the limitations of the study, there are significant differences between the surface roughness of the samples exposed to Pepsi and Mirinda on the third day. However, the Miranda sample showed the roughest surface. There is a statistically significant difference between the control and test samples (Pepsi and Mirinda) at 15 days of exposure, with both showing similar roughness.

## Data availability

Harvard Dataverse: Evaluation of the effect of soft drinks on the surface roughness of dental enamel in natural human teeth. https://doi.org/10.7910/DVN/VJC52A. ^18^

This project contains the following underlying data:

• data sheet.tab (profilometer measurement results)

Data are available under the terms of the Creative Commons Zero “No rights reserved” data waiver (CC0 1.0 Public domain dedication).
