# Supplementary material for: Evaluation of the effect of soft drinks on the surface roughness of dental enamel in natural human teeth
Source: F1000Res. 2021 Nov 10;10:1138. [Version 1] doi: 10.12688/f1000research.55556.1 (PMC8829096; doi:10.12688/f1000research.55556.1)
Supplement: Peer review history

# Peer review: Reviewer response for version 1

**DOI:** 10.5256/f1000research.59142.r99681

**Published:** 2022-02-09

**Authors:** Manuel S. Thomas

**Affiliations:** 1 Department of Conservative Dentistry and Endodontics, Manipal College of Dental Sciences, Manipal Academy of Higher Education, Mangalore, Karnataka, India

Changes in dietary habits, especially increased consumption of acidic food and drinks have been attributed to a steep increase in the prevalence of dental erosion over the years. ^1^ This article reinforces the fact that carbonated drinks can cause enamel erosion, especially when consumed for a prolonged period. Therefore, it is imperative that oral health care providers recognize the dietary pattern of their patients and give them sound oral care instructions. ^2^

 Apart from the pH of the drink, other aspects like dissociation constant (pKa), titratable acidity, buffering capacity, the concentration of calcium, phosphate, and fluoride ions as well as the degree of saturation of the product are also significant factors determining the erosive potential of the drink. ^3^ In addition to measuring the surface roughness of the enamel, other aspects such as weight loss, degree and depth of demineralization, and surface hardness will shed light on the destructive nature of dietary products on enamel. Accordingly, these topics could be considered as areas of potential future research.

 In this study 3D non-contact surface metrology was used to measure the enamel surface roughness. This is a non-damaging quantitative technique, unlike contact methods like stylus surface profilometer. Therefore, the method used in this study is ideal for measuring repeated values in the same area. [ref 4]. Another point to note in this study is that the enamel surface was exposed to the beverage for a prolonged period of time (up to 15 days). This longer duration of exposure can provide more insight into the effects of frequent consumption of these beverages on the enamel surface hardness.

 There are two spelling errors that I have highlighted in the pdf (page no. 6, paragraphs 4 and 5). I have also mentioned a couple of suggestions in the PDF [these are highlighted in yellow].

Is the work clearly and accurately presented and does it cite the current literature?

Yes

If applicable, is the statistical analysis and its interpretation appropriate?

Yes

Are all the source data underlying the results available to ensure full reproducibility?

Yes

Is the study design appropriate and is the work technically sound?

Yes

Are the conclusions drawn adequately supported by the results?

Yes

Are sufficient details of methods and analysis provided to allow replication by others?

Yes

Reviewer Expertise:

dental materials, restorative dentistry

I confirm that I have read this submission and believe that I have an appropriate level of expertise to confirm that it is of an acceptable scientific standard.

## References

[1] : Contemporary diagnosis and management of dental erosion. *J Esthet Restor Dent* .2021;33(1) : 10.1111/jerd.12706 78-87 10.1111/jerd.12706 33410255

[2] : Chapter 9: Acidic Beverages and Foods Associated with Dental Erosion and Erosive Tooth Wear. *Monogr Oral Sci* .2020;28: 10.1159/000455376 91-98 10.1159/000455376 31940633

[3] : Analysis of the erosive effect of different dietary substances and medications. *Br J Nutr* .2012;107(2) : 10.1017/S0007114511002820 252-62 10.1017/S0007114511002820 21733310

[4] : Roughness and pH changes of enamel surface induced by soft drinks in vitro-applications of stylus profilometry, focus variation 3D scanning microscopy and micro pH sensor. *Dent Mater J* .2011;30(3) : 10.4012/dmj.2010-204 404-10 10.4012/dmj.2010-204 21597209

# Peer review: Reviewer response for version 1

**DOI:** 10.5256/f1000research.59142.r99678

**Published:** 2022-02-09

**Authors:** Khalifa Sulaiman Al-Khalifa

**Affiliations:** 1 Department of Preventive Dental Sciences, College of Dentistry, Imam Abdulrahman Bin Faisal University, Dammam, Saudi Arabia

This study investigated the effect of soft drinks on the surface roughness of dental enamel in natural human teeth. The topic is interesting, and the manuscript is well written. The introduction covered the main ideas investigated in the study. All data of surface roughness are available, and the discussion explained the results thoroughly.

 However, some minor comments are suggested for the authors to consider:

 In the discussion, give a brief account of the limitations of your study.

 One of the limitations is the sample size which may not be optimal. Adequate sample size will result in more reliable, valid, and generalizable results. The authors also did not mention sample size/power calculation: determining the optimal sample size for a study assures an adequate power to detect statistical significance. If study is underpowered, it will be statistically inconclusive and may make the whole protocol a failure.

Is the work clearly and accurately presented and does it cite the current literature?

Yes

If applicable, is the statistical analysis and its interpretation appropriate?

Yes

Are all the source data underlying the results available to ensure full reproducibility?

Yes

Is the study design appropriate and is the work technically sound?

Yes

Are the conclusions drawn adequately supported by the results?

Yes

Are sufficient details of methods and analysis provided to allow replication by others?

Yes

Reviewer Expertise:

Cariology, Dental Public Health, Quality of life

I confirm that I have read this submission and believe that I have an appropriate level of expertise to confirm that it is of an acceptable scientific standard.
